# *Pochonia chlamydosporia* Induces Plant-Dependent Systemic Resistance to *Meloidogyne incognita*

**DOI:** 10.3389/fpls.2019.00945

**Published:** 2019-08-13

**Authors:** Zahra Ghahremani, Nuria Escudero, Ester Saus, Toni Gabaldón, F. Javier Sorribas

**Affiliations:** ^1^Departament d’Enginyeria Agroalimentària i Biotecnologia, Universitat Politècnica de Catalunya, Barcelona, Spain; ^2^Bioinformatics and Genomics Programs, Centre for Genomic Regulation (CRG), Barcelona Institute of Science and Technology, Barcelona, Spain; ^3^Department of Experimental and Health Sciences, Universitat Pompeu Fabra (UPF), Barcelona, Spain; ^4^ICREA, Barcelona, Spain

**Keywords:** *Cucumis sativus*, induced resistance, root endophytes, root-knot nematodes, *Solanum lycopersicum*

## Abstract

*Meloidogyne* spp. are the most damaging plant parasitic nematodes for horticultural crops worldwide. *Pochonia chlamydosporia* is a fungal egg parasite of root-knot and cyst nematodes able to colonize the roots of several plant species and shown to induce plant defense mechanisms in fungal-plant interaction studies, and local resistance in fungal-nematode-plant interactions. This work demonstrates the differential ability of two out of five *P. chlamydosporia* isolates, M10.43.21 and M10.55.6, to induce systemic resistance against *M. incognita* in tomato but not in cucumber in split-root experiments. The M10.43.21 isolate reduced infection (32–43%), reproduction (44–59%), and female fecundity (14.7–27.6%), while the isolate M10.55.6 only reduced consistently nematode reproduction (35–47.5%) in the two experiments carried out. The isolate M10.43.21 induced the expression of the salicylic acid pathway (*PR-1* gene) in tomato roots 7 days after being inoculated with the fungal isolate and just after nematode inoculation, and at 7 and 42 days after nematode inoculation too. The jasmonate signaling pathway (*Lox D* gene) was also upregulated at 7 days after nematode inoculation. Thus, some isolates of *P. chlamydosporia* can induce systemic resistance against root-knot nematodes but this is plant species dependent.

## Introduction

The root-knot nematodes (RKN), *Meloidogyne* spp., are obligate parasites of plants. The genus comprises more than 100 species, but only four of them are considered the most damaging plant parasitic nematodes due to its wide range of plant hosts, worldwide distribution, and high reproductive capacity (Jones et al., 2013). The RKN infective juveniles (J2) enter the root near the elongation zone and migrate intercellularly to establish a permanent feeding site into the vascular cylinder, inducing the formation of giant cells and root galls by affecting cell wall architecture, plant development, defenses, and metabolism (Shukla et al., 2018). Once the infection occurs, J2 become sedentary, and molt three times to achieve the mature adult female stage. The most frequent and damaging tropical species, *M. arenaria*, *M. incognita*, and *M. javanica*, reproduce parthenogenetically. The female lays a large number of eggs in a gelatinous matrix, the egg mass, located on the surface or within the galled roots.

The damage potential of some *Meloidogyne* species has been summarized (Greco and Di Vito, 2009; Wesemael et al., 2011). *M. arenaria*, *M. incognita* and *M. javanica* are responsible for the majority of vegetable yield losses caused by plant parasitic nematodes (Sikora and Fernández, 2005). Among vegetables, those belonging to the solanaceae and cucurbitaceae families are commonly included in rotation schemes because they are economically important for growers. The estimation of maximum crop yield losses caused by the nematode in field and plastic greenhouse cultivation varies according to the plant germplasm, environmental conditions and agronomic practices. For instances, maximum yield losses from 62 to 100% have been reported in susceptible tomato cultivars (Seid et al., 2015; Giné and Sorribas, 2017), 30 to 60% in aubergine (Sikora and Fernández, 2005), 50% in cantaloupe (Sikora and Fernández, 2005), 37 to 50% in watermelon (Sikora and Fernández, 2005; López-Gómez et al., 2014), and 88% in non-grafted or grafted cucumber on *Cucurbita* hybrid rootstocks (Giné et al., 2014, 2017). Control of RKN is conducted mainly with fumigant and non-fumigant nematicides (Djian-Caporalino, 2012; Talavera et al., 2012). However, due to environmental and toxicological concerns, some legislative regulations, such as the European Directive 2009/128/EC, aim to reduce the use of pesticides by promoting alternative methods such as biological control and plant resistance.

Several nematode antagonists belonging to different taxonomic groups have been described (Stirling, 2014). They can act in three ways: (1) directly parasitizing several or specific RKN development stages, such as *Pasteuria penetrans* (Davies et al., 2011); (2) indirectly by repelling, immobilizing and/or killing them by means of metabolites, and/or inducing plant response, such as *Fusarium oxysporum* strain Fo162 (Dababat and Sikora, 2007a,b); or (3) both directly and indirectly, such as *Trichoderma atroviride* strain T11 or *Trichoderma harzianum* strain T-78 (de Medeiros et al., 2017; Martínez-Medina et al., 2017). *Pochonia chlamydosporia* (syn. *Metacordyceps chlamydosporia*) is a fungal antagonist of RKN and cyst nematodes that acts directly by parasitizing eggs, and could also acts indirectly. This fungal species colonizes endophytically the root of several plants, including barley (Maciá-Vicente et al., 2009), tomato (Bordallo et al., 2002), potato (Manzanilla-López et al., 2011), or *Arabidopsis* (Zavala-Gonzalez et al., 2017), inducing plant defense mechanisms, such as the formation of papillae (Bordallo et al., 2002) and the modulation of miRNA in tomato (Pentimone et al., 2018) or plant defense genes related to salicylic acid and jasmonic acid pathways in barley and *Arabidopsis* (Larriba et al., 2015; Zavala-Gonzalez et al., 2017). It is assumed that some of these defense mechanisms could suppress root infection, development and/or reproduction of RKN (Escudero and Lopez-Llorca, 2012), but as far we know, only one study has proven the induction of local resistance (de Medeiros et al., 2015), and none to elucidate the capability of this fungal species to induce systemic resistance. As *P. chlamydosporia* parasitizes RKN eggs, a split-root system is required to determine the capability of this nematophagous fungus to induce plant resistance avoiding the direct interaction with the nematode. Therefore, in the present study, the capability of five *P. chlamydosporia* isolates to induce plant resistance against *M. incognita* was assessed in a split-root system. To assess whether the response was plant dependent, tomato and cucumber were used as representatives of solanaceous and cucurbit crops frequently including in rotation schemes.

## Materials and Methods

### Plant Material, Nematode, and Fungi

Tomato cv. Durinta and cucumber cv. Dasher II were used in this study. For all the experiments, seeds were surface-sterilized in a 50% sterilized bleach solution (35 g L^−1^ active chlorine) for 2 min, washed three times in sterilized distilled water for 10 s each, sown in a tray containing sterile vermiculite, and maintained in a growth chamber at 25°C ± 2°C with a 16 h:8 h (light:dark) photoperiod.

Five *P. chlamydosporia* isolates M10.41.42, M10.43.21, M10.51.3, M10.55.6, and M10.62.2 were used. The fungal isolates were obtained from horticultural commercial growing sites in northeastern Spain from RKN eggs (Giné et al., 2012) and maintained as single-spore isolates at the Universitat Politècnica de Catalunya. Fungal chlamydospores were produced in barley seeds following the procedure of Becerra and collaborators (Becerra Lopez‐Lavalle et al., 2012) with some modifications. Briefly, for each isolate, three 200 g batches of barley seeds were soaked for 18 h and each batch sterilized in an Erlenmeyer flask at 121°C for 22 min over two consecutive days, then were incubated at 25°C ± 2°C in the dark. Afterward, 10 5-mm plugs from the edge of each *P. chlamydosporia* isolate grown in CMA were added to each Erlenmeyer flask and they were shaken at 5-day intervals to homogenize fungal growth. After a month, the number of chlamydospores produced on barley was determined following the procedure of Kerry and Bourne (2002). Three 10-seed subsamples per Erlenmeyer were plated onto CMA and incubated at 25 ± 2°C in the dark for 2 weeks to assess putative contaminations prior to being used. The viability of the chlamydospores was assessed as in Escudero et al. (2017).

J2 of the isolate Agropolis of *Meloidogyne incognita* were used as inoculum. Eggs were extracted from tomato roots by blender maceration in a 5% commercial bleach (40 g L^−1^ NaOCl) solution for 5 min (Hussey and Barker, 1973). The egg suspension was passed through a 74-μm aperture sieve to remove root debris, and eggs were collected on a 25-μm sieve and placed on Baermann trays (Whitehead and Hemming, 1965) at 25 ± 2°C. Nematodes were collected daily using a 25-μm sieve for 7 days and stored at 9°C until their use.

### Induction of Systemic Plant Resistance by *P. chlamydosporia* Isolates Against Root-Knot Nematodes

Tomato and cucumber were grown in a split-root system as described in previous studies (de Medeiros et al., 2017; Martínez-Medina et al., 2017). In this system, the root is divided into two halves transplanted in two adjacent pots: the inducer, inoculated with the antagonist, and the responder, inoculated with the nematode. Briefly, the main root of 5-day-old seedlings was excised and plantlets were individually transplanted in seedling trays containing sterile vermiculite and maintained under the same conditions for 2 weeks for cucumber, and 3 weeks for tomato plants. Afterward, plantlets were transferred to the split-root system by splitting roots into two halves planted in two adjacent 200 cm^3^ pots filled with sterilized sand. Four treatments were assessed for each fungal isolate: Fungi-RKN and None-RKN, to assess the capability of each fungal isolate to induce plant response against RKN, and Fungi-None and None-None, to assess the effect of each fungal isolate on plant growth. Each treatment was replicated 10 times, and the experiment was conducted two times. The inducer part of the root of the treatments containing Fungi was inoculated with 10^5^ viable chlamydospores of *P. chlamydosporia* just before transplanting. One week later, the responder part of the root of the treatments containing RKN was inoculated at a rate of 1 J2 per cm^3^ of soil. The treatment None-None, with no inoculation with either fungi or RKN received the same volume of water. The plants were maintained in a growth chamber in the same conditions described previously in a completely randomized design for 40 days. The plants were irrigated as needed and fertilized with Hoagland solution twice per week. Soil temperatures were recorded daily at 30-min intervals with a PT100 probe (Campbell Scientific Ltd) placed in the pots at a depth of 4 cm. At the end of the experiments, both inducer and responder root fresh weight and the shoot dry weight of each single plant were measured. Roots from the RKN-inoculated responder were immersed in a 0.01% erioglaucine solution for 45 min to stain the egg masses (Omwega et al., 1988) before counting them. Afterward, the eggs were extracted from the roots as in Hussey and Barker (1973)’s method and counted. The number of egg masses was considered as the infective capability of the nematode because it indicates the number of J2 able to penetrate, to infect the root, and to develop into egg-laying females. The number of eggs was considered the reproductive capability of the nematode, and the female fecundity was calculated as the number of eggs per egg mass.

The tomato and cucumber root colonization by each fungal isolates was estimated by quantifying the fungal DNA by qPCR at the end of the second experiment. The inducer part of the root was washed three times in sterilized distilled water for 10 s each and then blotted onto sterile paper. Per each fungal isolate three biological replicates were assessed. Each biological replicate consisted of the inducer part of the roots from three plants pooled together. The DNA was extracted from each biological replicate following the Lopez-Llorca et al. (2010)’s procedure. qPCR reactions were performed using the FastStart Universal SYBR Green Master (Roche) mix in a final volume of 25 μl containing 50 ng of total DNA and 0.3 μM of each primer (5′ to 3′ direction) VCP1-1F (CGCTGGCTCTCTCACTAAGG) and VCP1-2R (TGCCAGTGTCAAGGACGTAG) (Escudero and Lopez-Llorca, 2012). Negative controls containing sterile water instead of DNA were included. Reactions were performed in duplicate in a Stratagene Mx3005P thermocycler (Agilent Technologies) using the following thermal cycling conditions: initial denaturation step at 95°C for 2 min, then 40 cycles at 95°C for 30 s, and 62°C for 30 s. Genomic DNA dilutions of the fungal isolate M10.43.21 were used to define a calibration curve from 5 pg to 50 ng. After each run, the specificity of the PCR amplicons was verified by melting curve analysis and agarose gel electrophoresis. The fungal DNA biomass of each isolate was referred to the total DNA biomass (50 ng) and expressed as a proportion.

### Dynamic Regulation of the Jasmonic and Salicylic Acid Pathways by *P. chlamydospora* and *M. incognita*

Tomato seeds were sterilized as previously described. Three-week-old tomato seedlings were transferred to 200 cm^3^ pots with sterilized sand and maintained in a growth chamber as previously described. The fungal isolate M10.43.21 was selected for this experiment because it reduced nematode infectivity and reproduction in the split-root system experiments. The experiment consisted of two treatments: non-inoculated and co-inoculated to determine the expression of genes related to the salicylic acid and jasmonic acid pathways. In the co-inoculated treatment, the soil was inoculated with 10^5^ viable chlamydospores just before transplanting and with 1 J2 of *M. incognita* per cm^3^ 1 week after transplanting. Each treatment was replicated 40 times. An additional treatment only inoculated with the nematode was included to determine the effect of the fungal isolate on nematode reproduction. The fungus and nematode inoculation procedure was as previously stated.

The expression of the pathogenesis-related protein 1 (*PR-1*) gene and the lipoxygenase (*Lox D*) gene the from the salicylic acid and jasmonic acid pathways, respectively, was evaluated at three time points: just after nematode inoculation, that is, at 0 days after nematode inoculation (dani), at 7 dani, and at 42 dani. At each assessment time, roots were washed three times with sterile distilled water, placed onto sterilized filter paper, frozen in liquid nitrogen and stored at −80°C until being used. At 7 dani, the J2 were stained inside roots with acid Fuchsin following the Byrd et al. (1983) procedure to confirm that nematode had penetrated and infected. At the end of the experiment (42 dani), the number of eggs per plant from three plants for each treatment was determined by extracting them as described previously.

Total RNA from roots was isolated using the PureLink RNA Mini Kit (Invitrogen), according to the manufacturer’s instructions. Afterward, the DNA-free kit (Invitrogen) was used to remove the remaining DNA from the sample. Total RNA integrity and quantity of the samples were assessed by means of agarose gel, NanoDrop 1000 Spectrophotometer (Thermo Scientific) and Qubit RNA BR assay kit (Thermo Fisher Scientific). To assure that the sample was DNA free, a PCR was carried out. Then, the RNA was retro-transcribed with the SuperScript II (Invitrogen) according to manufacturer’s instructions. The relative gene expression was estimated with the ∆∆Ct methodology (Livak and Schmittgen, 2001), using the ubiquitin (*UBI*) gene as a reference gene (Song et al., 2015). Primers used in the RT-qPCR were (5′ to 3′ direction): LeUbi3-F (TCCATCTCGTGCTCCGTCT), LeUbi3-R (GAACCTTTCCAGTGTCATCAACC) Song et al. (2015), LoxD-F (GACTGGTCCAAGTTCACGATCC), LoxD-R (ATGTGCTGCCAATATAAATGGTTCC) Fujimoto et al. (2011), LEPR1F (GCAACACTCTGGTGGACCTT), and LEPR1R (ATGGACGTTGTCCTCTCCAG) Gayoso et al. (2007). qPCR reactions were performed in a final volume of 20 μl with 1 μl of cDNA, 0.3 mM of the corresponding primers, and 1X Fast SYBR Green Master Mix (Applied Biosystems). The qPCR was performed in a 7900HT Fast Real Time PCR System thermocycler (Applied Biosystems) using: 20 s at 95°C followed by 40 cycles of 30 s at 95°C and 60 s at 60°C (Gayoso et al., 2007). The specificity of PCR amplicons was verified as described previously. Reactions were performed with three biological replicates per treatment. Each biological replicate consisted of the roots from three plants pooled together. Two technical replicates per biological replicate were assessed.

### Statistical Analysis

Statistical analyses were performed using the JMP software v8 (SAS institute Inc., Cary, NC, USA). Both data normality and homogeneity of variances were assessed. When confirmed, a paired comparison using the Student’s *t*-test was done. Otherwise, paired comparison was done using the non-parametric Wilcoxon test, or multiple comparison using the Kruskal-Wallis test and groups separated by Dunn’s test (*p* ≤ 0.05). The repetitions of the split-root experiments for each crop were compared using the non-parametric Wilcoxon test, and considered as one experiment when no differences (*p* ≤ 0.05) were found.

## Results

### Induction of Systemic Plant Resistance by *P. chlamydosporia* Isolates Against *M. incognita*

The split-root system experiments with tomato differed (*p* < 0.05) between them and were treated separately. But no differences were found between the two split-root experiments with cucumber and thus we considered them as replicates of a single experiment. Both tomato and cucumber fresh root weight of the two halves of the split-root system of the None-None treatment did not differ (*p* < 0.05) (data not shown), showing that the split-root system did not influence root development. Shoot dry biomass did not differ in any fungal isolate-plant species combination (*p* < 0.05) (data not shown).

Two of the five *P. chlamyodosporia* isolates induced resistance in tomato plants in both experiments, but none of them did in cucumber (Table 1). The fungal isolate M10.43.21 reduced both the number of egg masses per plant (32–43%), the number of eggs per plant (44–59%), and the female fecundity (14.7–27.6%), while the isolate M10.55.6 reduced the number of eggs per plant in both experiments (35–47.5%) but the number of egg masses or the female fecundity in only one.

**Table 1:** Capability of *P. chlamydosporia* isolates to induce systemic resistance in tomato cv. Durinta or cucumber cv. Dasher II against *Meloidogyne incognita* in two split root experiments.

Crop-experiment	Fungal isolate	Egg masses per plant	Eggs (×10^3^) per plant	Eggs per egg mass
Tomato – 1	M10.41.42	110 ± 14	56.6 ± 8.3	517 ± 41
	M10.43.21	67 ± 16^*^	29.5 ± 7.3^*^	445 ± 41^*^
	M10.51.3	91 ± 5	45.1 ± 3.2^*^	500 ± 27^*^
	M10.55.6	82 ± 9^*^	38.0 ± 6.1^*^	506 ± 102
	M10.62.2	103 ± 8	44.3 ± 2.4^*^	434 ± 16^*^
	Non-inoculated	118 ± 5	72.4 ± 3.3	615 ± 31
Tomato – 2	M10.41.42	71 ± 4	62.1 ± 4.4	876 ± 37
	M10.43.21	57 ± 7^*^	44.4 ± 5.3^*^	812 ± 68^*^
	M10.51.3	65 ± 7	58.9 ± 8.1	881 ± 40
	M10.55.6	62 ± 9	51.3 ± 7.6^*^	831 ± 28^*^
	M10.62.2	68 ± 5	60.4 ± 3.8	897 ± 38
	Non-inoculated	84 ± 7	79.3 ± 7.1	952 ± 41
Cucumber – 1 and 2	M10.41.42	52 ± 6	24.9 ± 4.3	459 ± 42
	M10.43.21	52 ± 5	24.7 ± 3.0	464 ± 22
	M10.51.3	54 ± 6	26.1 ± 3.6	466 ± 28
	M10.55.6	53 ± 7	25.4 ± 5.2	463 ± 40
	M10.62.2	51 ± 6	24.8 ± 4.4	458 ± 39
	Non-inoculated	46 ± 6	20.5 ± 4.2	422 ± 45

*The inducer part of the root was inoculated with 10^5^ chlamydospores of the fungus just before transplanting and the responder part of the root was inoculated with 200 nematode juveniles a week later. The parameters assessed in the responder part of the root at the end of the experiments, 40 days after being inoculated with the nematode, were: the number of egg masses per plant (infectivity), the number of eggs per plant (reproduction), and the number of eggs per egg mass (fecundity)*.

Data of each tomato experiment are mean ± SE of 10 replicates. Data of the cucumber experiments 1 and 2 are mean ± SE of 20 replicates because no differences (p < 0.05) were found between experiments and data were considered as a single experiment. Data within the same column per crop and experiment followed by ^*^differ (p < 0.05) from the non-inoculated treatment according to the non-parametric Wilcoxon test.

**Figure 1:**
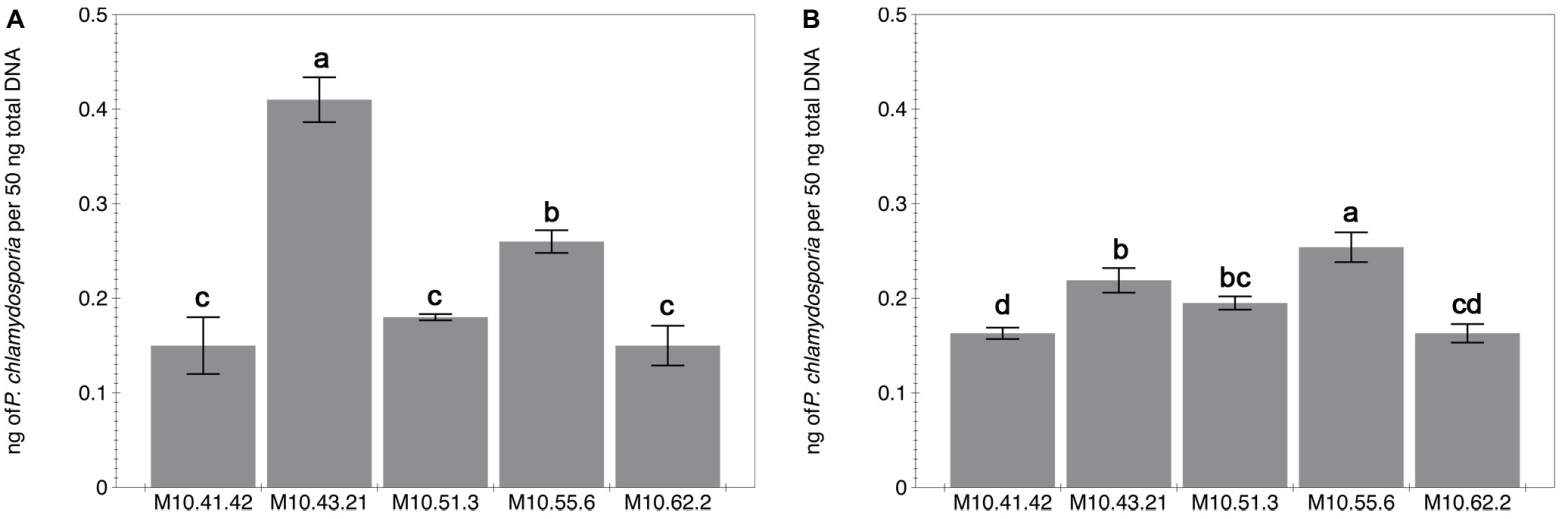
Capability of five *P. chlamydosporia* isolates to colonize roots of tomato cv. Durinta **(A)** and cucumber cv. Dasher II **(B)**. The root colonization is expressed as the proportion of fungal DNA biomass (isolates) per 50 ng of the total DNA biomass extracted from the inducer part of the root of the split-root experiment 2. Each value is mean ± SE of three biological samples with two technical replicates each. Different letters indicate statistical differences between isolates (*p* < 0.05) according to the Dunn’s test.

*P. chlamydosporia* isolates differed in the level of root colonization estimated by qPCR irrespective of the plant species (Figure 1). The standard curves for qPCR obtained by representing the cycle thresholds (Ct) against the log of 10-fold serial dilution of DNA from isolate M10.43.21 were accurate and reproducible to estimate the DNA concentration of the different treatments (tomato = −3.66*x* + 19.36; *R*^2^ = 0.9736 and cucumber *y* = −3.4937*x* + 21.29; *R*^2^ = 0.9947). Regarding tomato, isolate M10.43.21 was the best root colonizer followed by M10.55.6 (Figure 1A). In relation to cucumber, isolate M10.55.6 was the best root colonizer followed by M10.43.21 (Figure 1B). The fungus was not detected in the inducer part of the root from treatments None-None and None-RKN.

### Dynamic Regulation of the Jasmonic and Salicylic Acid Pathways by *P. chlamydosporia* and *M. incognita* in Tomato

**Figure 2:**
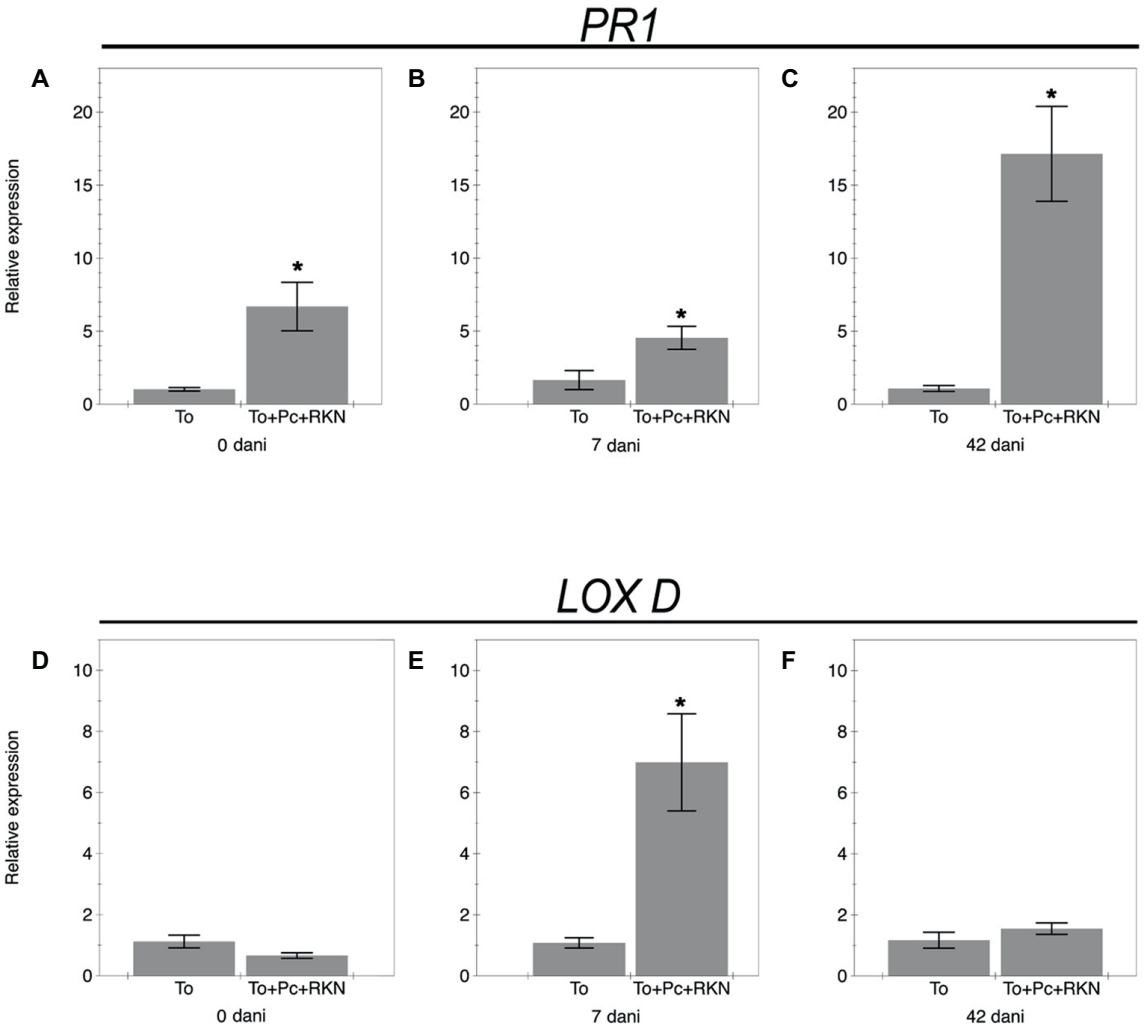
Relative expression of genes related to the salicylic acid and jasmonic acid pathways. The expression of genes *Pr1* **(A–C)** and *Lox D* **(D–F)** in roots of the tomato cv. Durinta non inoculated (To) or inoculated with 10^5^ chlamydospores of *P. chlamydosporia* isolate M10.43.21 just before transplanting and with 200 J2 of *M. incognita* per plant a week after transplanting (To+Pc + RKN) at three time points: just after nematode inoculation (0 dani), at 7 dani, and at 42 dani. Each value is mean ± SE of three biological samples with two technical replicates each. Asterisks indicate significant differences (*p* < 0.05) according to the non-parametric Wilcoxon test.

Changes in the expression of genes *PR-1* and *Lox D* from the salicylic acid and jasmonic acid pathways at 0, 7, and 42 dani are shown in Figure 2. The expression of the *PR-1* gene in roots inoculated with the fungal isolate M10.43.21 was upregulated at 0, 7, and 42 dani compared to the non-inoculated plants (Figures 2A–C). Regarding the jasmonic acid pathway (Figures 2D–F), the gene *Lox D* was only upregulated at 7 dani.

**Figure 3:**
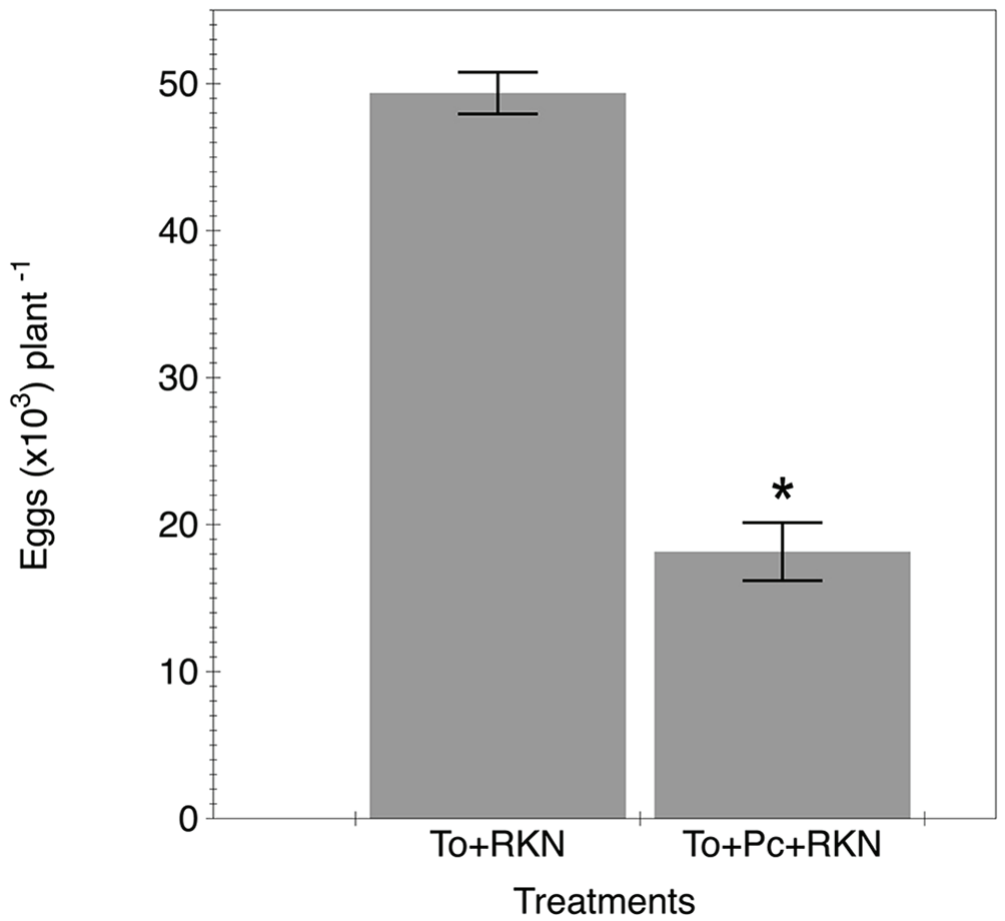
Effect of primed tomato plants by *P. chamydosporia* on *M. incognita* reproduction. Number of eggs produced in the tomato cv. Durinta after 42 days of being inoculated with 200 J2 of *M. incognita* per plant a week after transplanting (To + RKN) or inoculated with 10^5^ chlamydospores of *P. chlamydosporia* isolate M10.43.21 just before transplanting and with the nematode at the same rate and time mentioned before (To + Pc + RKN). Each value is mean ± SE of three replications. Asterisk indicates significant differences (*p* < 0.05) according to the non-parametric Wilcoxon test.

The nematode reproduction in plants co-inoculated with the fungal isolate was suppressed by 60% compared to plants only inoculated with the nematode (Figure 3).

## Discussion

The results of this study provide evidence for the ability of some *P. chlamydosporia* isolates to induce systemically resistance against *M. incognita*, and that this induction is dependent on the plant species. The isolate M10.43.21 showed the most consistent response in both split-root experiments with tomato, and was the reason for selecting it to determine the hormone modulation in this plant species. The mechanisms responsible for the endophyte-induced resistance are unclear (Schouten, 2016). Both salicylic acid- and jasmonic acid-dependent signaling pathways have been proposed as responsible for the systemically induced resistance to *Meloidogyne* spp. in tomato in split-root experiments (Selim, 2010; de Medeiros et al., 2017; Martínez-Medina et al., 2017). Martínez-Medina et al. (2017) reported that *Trichoderma harzianum* T-78 induced the upregulation of genes related to salicylic acid at early stage of nematode infection, whereas those related to jasmonic acid were upregulated from 3 to 21 days after nematode inoculation. In our study, the *P. chlamydosporia* isolate primed salicylic acid from the first assessment time (7 days after fungal inoculation and just after nematode inoculation) until the end of the experiment (42 dani). This effect could be responsible for the suppression of nematode infection at early stages, as well as the infection by the J2 produced by the primary inoculum that were able to overcome the plant defense mechanisms. In addition, the upregulation of the *Lox D* gene, related to jasmonic acid, at 7 dani could affect nematode reproduction and fecundity. Thus, the induction of the salicylic acid and jasmonic acid signaling pathways by the fungal isolate M10.43.21 in tomato counteract the suppression of these phytohormones by the nematode described in the susceptible tomato-nematode interaction (Shukla et al., 2018). The three-phase model proposed to explain the induced protection to RKN by *Trichoderma harzianum* T-78 in tomato consisting of salicylic acid induction suppressing RKN infection followed by jasmonic acid induction suppressing RKN reproduction and fecundity and finally salicylic acid induction affecting root infection by the next J2 generation (Martínez-Medina et al., 2017) is valid for *P. chlamydosporia.* Other local plant defense mechanisms induced by *P. chlamydosporia* against RKN have been reported, including the increase of the peroxidases (*POX*) and poliphenoloxidases (*PPO*) enzymes activity at root nematode invasion stage (24–96 h after nematode inoculation) (de Medeiros et al., 2015). However, considering that *P. chlamydosporia* does not extensively colonize the root (Maciá-Vicente et al., 2009; Escudero and Lopez-Llorca, 2012), even being improved by chitosan irrigation (Escudero et al., 2017), the effect of local defense mechanisms alone may be insufficient to achieve significant nematode suppression.

Not all *P. chlamydosporia* isolates induced systemic resistance in tomato. The variability of this attribute among fungal isolates can be added to other observations previously reported, such as the production of chlamydospores, root colonization, plant growth promotion, or egg parasitism (Kerry and Bourne, 2002; Zavala-Gonzalez et al., 2015). In fact, egg parasitism can even differ between single-fungal spore isolates originating from the same field population (Giné et al., 2016). The frequency of occurrence of *P. chlamydosporia* in horticultural production sites under integrated and organic standards has increased since the 1990s in northeastern Spain (Giné et al., 2012), showing that this fungal species is adapted to environmental characteristics and agronomic practices. Field populations of *P. chlamydosporia* can contain individuals representing a diversity of functions that are highly beneficial to plants, such as plant growth promoters which enhance plant tolerance; inducers of plant defense mechanisms suppressing infection, development and reproduction of RKN; efficient egg parasites suppressing the RKN inoculum; or saprophytic behavior contributing to the organic matter cycle and plant nutrition. A given proportion of *P. chlamydosporia* representing some or all of these functions could be present in a given soil and adapted to the plant species involved in the rotation scheme and contributing to their health status. It seems that most of these functions are not interlinked. In fact, none of the five fungal isolates assessed in our study was a plant growth promoter. Thus, molecular tools must be developed to facilitate knowledge of the functional composition of the fungal field population in a given soil.

*P. chlamydosporia* has been reported as the main biotic factor responsible for soil suppressiveness to RKN in horticultural crops (Giné et al., 2016). In soils with low antagonistic potential, the use of fungal isolates with both direct and indirect action mechanisms could suppress RKN. Indeed, primed plants along with egg parasitism will protect against infection and reproduction of RKN and decrease the inoculum viability. The combination of the two modes of action will result in a decrease of the nematode population growth rate, and consequently lower crop yield losses. Alternatively, combining the use of *P. chlamydosporia* with plant defense activators can produce a similar effect. Vieira Dos Santos et al. (2014) found a reduction in RKN reproduction when *P. chlamydosporia* was combined with the application of cis-jasmone, as well as an increase in fungal egg parasitism.

In conclusion, this study proves that some *P. chlamydosporia* isolates induce systemic resistance to *M. incognita* in tomato but none of them in cucumber. Thus, this response is plant species dependent. In future studies, the interaction between *P. chlamydosporia* isolates and selected economically important crops should be characterized to elucidate the mechanisms and genes involved in inducing plant resistance in order to maximize the efficacy of control.

## Data Availability

All datasets generated for this study are included in the manuscript and/or the supplementary files.

## Author Contributions

FS and NE conceived, designed, supervised the experiments, the data collection, and analyses. ZG performed the experiments, analyzed the data, and wrote the draft of the manuscript. NE and ES performed the gene expression analyses. TG provided reagents, materials, and advice. NE, ES, TG, and FS reviewed and wrote the final draft of the manuscript.

### Conflict of Interest Statement

The authors declare that the research was conducted in the absence of any commercial or financial relationships that could be construed as a potential conflict of interest.
